# Time Trends of Environmental and Socioeconomic Risk Factors in Patients with Inflammatory Bowel Disease over 40 Years: A Population-Based Inception Cohort 1977–2020

**DOI:** 10.3390/jcm12083026

**Published:** 2023-04-21

**Authors:** Panu Wetwittayakhlang, Lorant Gonczi, Petra A. Golovics, Zsuzsanna Kurti, Tunde Pandur, Gyula David, Zsuzsanna Erdelyi, Istvan Szita, Laszlo Lakatos, Peter L. Lakatos

**Affiliations:** 1 Division of Gastroenterology and Hepatology, McGill University Health Centre, Montreal, QC H3G 1A4, Canada; wet.panu@gmail.com; 2 Gastroenterology and Hepatology Unit, Division of Internal Medicine, Faculty of Medicine, Prince of Songkla University, Hat Yai 90110, Thailand; 3 Department of Internal Medicine and Oncology, Semmelweis University, 1083 Budapest, Hungary; lorantgonczi@gmail.com (L.G.); zsuzsa.kurti@gmail.com (Z.K.); 4 Department of Gastroenterology, Hungarian Defence Forces Medical Centre, 1062 Budapest, Hungary; golovicspetra@gmail.com; 5 Department of Gastroenterology, Grof Eszterhazy Hospital, 8500 Papa, Hungary; pandur.tunde.dr@gmail.com; 6 Department of Gastroenterology, Ferenc Csolnoky Hospital, 8200 Veszprem, Hungary; david.gyula@gmail.com (G.D.); erdelyi.zsuzsanna.dr@gmail.com (Z.E.); szitaistvan1@gmail.com (I.S.);

**Keywords:** inflammatory bowel disease, ulcerative colitis, Crohn’s disease, risk factor, environmental, socioeconomic, smoking, contraceptive use, appendectomy

## Abstract

Background: Data from population-based studies investigating trends in environmental factors associated with inflammatory bowel disease (IBD) is lacking. We aimed to assess long-term time trends of environmental and socioeconomic factors in IBD patients from a well-defined population-based cohort from Veszprem, Hungary. Methods: Patients were included between 1 January 1977, and 31 December 2020. Trends of environmental and socioeconomic factors were evaluated in three periods based on the decade of diagnosis, representing different therapeutic eras: cohort-A,1977–1995; cohort-B,1996–2008 (immunomodulator era); and cohort-C, 2009–2020 (biological era). Results: A total of 2240 incident patients with IBD were included (ulcerative colitis (UC) 61.2%, male 51.2%, median age at diagnosis: 35 years (IQR 29–49)). Rates of active smoking significantly decreased over time in Crohn’s disease (CD): 60.2%, 49.9%, and 38.6% in cohorts A/B/C (*p* < 0.001). In UC, the rates were low and stable: 15.4%, 15.4%, and 14.5% in cohorts A/B/C (*p* = 0.981). Oral contraceptive use was more common in CD compared to UC (25.0% vs. 11.6%, *p* < 0.001). In UC, prevalence of appendectomy before diagnosis decreased over time: 6.4%, 5.5%, and 2.3% in cohorts A/B/C (*p* = 0.013). No significant changes were found in the socio-geographic characteristics of the IBD population (urban living: UC, 59.8%/64.8%/ 62.5% (*p* = 0.309) and CD, 62.5%/ 62.0%/ 59.0% (*p* = 0.636), in cohorts A/B/C). A greater percentage of patients had completed secondary school as the highest education level in later cohorts in both UC (42.9%/50.2%/51.6%, *p* < 0.001) and CD (49.2%/51.7%/59.5%, *p* = 0.002). A higher percentage of skilled workers (34.4%/36.2%/38.9%, *p* = 0.027) was found in UC, but not in CD (*p* = 0.454). Conclusion: The association between trends of known environmental factors and IBD is complex. Smoking has become less prevalent in CD, but no other major changes occurred in socioeconomic factors over the last four decades that could explain the sharp increase in IBD incidence.

## 1. Introduction

Ulcerative colitis (UC) and Crohn’s disease (CD) are chronic idiopathic inflammatory bowel diseases (IBD). The incidence of IBD has been highest in North America and Europe. In the 21st century, IBD has become a global disease with accelerating incidence in newly industrialized countries whose societies have become more westernized [1,2].

The pathogenesis of IBD is caused by a complex interaction of genetic and environmental factors [3]. Population-level changes in genetic susceptibility alone cannot explain the sharp increase in the incidence of IBD over the last few decades, since genetic changes do not take place within a short period of time. Thus, environmental factors are suggested to play an important role in the development of IBD [4,5,6]. The hygiene hypothesis suggests that the increased incidence of IBD reflects an alteration of immune responses, due to improvements in personal hygiene and reduced environmental antigenic exposure [5,7].

Multiple environmental factors associated with the risk for IBD have been identified [8,9]. Active smoking at diagnosis [4,10], oral contraceptive use [11], and previous appendectomy have been associated with increased risk of CD [12]. Previous smoking and oral contraceptive use have been linked with the development of UC, while both active smoking at the time of diagnosis [10] and appendectomy are protective factors in UC [13,14].

Several hygiene-related factors, such as childhood living area, education level, and personal lifestyle factors, may contribute to a higher risk of IBD [15]. Multiple studies have found a greater incidence of IBD in urban areas, indicating that growing up in an urban area is more hygienic than growing up in a rural area [16,17,18,19,20]. Increased physical activity is associated with a lower risk of CD [21,22]. Furthermore, occupations involving physically demanding activities are protective, whereas sedentary indoor occupations confer the risk of IBD [23]. Moreover, diet, tonsillectomy, antibiotic use, and allergies have been identified as risks for developing IBD [15,24].

Given the significant accelerated incidence of IBD in the last several decades, it is important to understand the magnitude of risk associated with the various environmental factors in the development of IBD. However, there has been a lack of data that could identify long-term trends of environmental factors in IBD, using population-based cohorts.

In the present study, we aimed to assess the long-term trends of environmental and socioeconomic factors in patients with IBD according to the different therapeutic eras using a well-established prospective population-based inception cohort of patients with IBD in Veszprem Province, western Hungary, between 1977 and 2020. 

## 2. Materials and Methods

### 2.1. Study Design and Population

This prospective population-based inception cohort study of patients with IBD was performed in Veszprem Province, a well-defined administrative region in the western part of Hungary. The study comprised consecutive individuals who were diagnosed with IBD and lived permanently in the investigated region between 1 January 1977 and 31 December 2020. 

Diagnoses of IBD (based on clinical symptoms, endoscopic, radiological, and histopathological evidence) generated in each hospital and outpatient unit were comprehensively examined using the Lennard-Jones and Copenhagen diagnostic criteria for each patient [25,26]. Only patients who met the eligibility criteria for CD and UC and had been diagnosed for more than one year were included in the analysis. Patients aged <18 years and those with undetermined colitis at the time of diagnosis were excluded from the study.

In addition, the permanent population and immigrant numbers in the Veszprém province were relatively stable between 1970 and 2020, with a total of 339,291 residents (updated on 1 January 2022), according to the reports of the Hungarian Central Statistical Office [27]. The province consists of both industrial and agricultural regions. Of the Veszprem population, 50.9% were in agricultural work. The ratio of urban/rural residents was also relatively stable (55% urban). The Romani population is lower than the Hungarian average (2.5%), while few people of Jewish ethnicity live in the region. 

The Veszprem Province database provided population-based data on the incidence of IBD from the 1970s. Previous studies from this region reported a sharply increasing incidence of CD: 0.4/10^5^ person-years (py) in the period 1977–1981, then 4.7/10^5^ py (1997–2001), 8.9/10^5^ py (2002–2006), and 9.9/10^5^ py (2007–2018) [28,29,30]. A similar phenomenon of increasing incidence was found initially for UC: 1.8/10^5^ py in the period 1977–1981, and 12.5/10^5^ py in the period 1997–2001. Thereafter, plateauing incidence rates were registered: 11.9/10^5^ py (2002–2006) and 11.0/10^5^ (2007–2018) [28,29,31].

This population-based inception cohort enabled assessment of the long-term trends of certain environmental and socioeconomic factors associated with the incidence of disease in the different therapeutic eras of IBD. In Hungary, thiopurines were first used to treat IBD in the mid-1990s, and anti-TNF therapy has been utilized and reimbursed by insurance since 2008. Therefore, the patients were divided into three consecutive cohorts based on the year of IBD diagnosis: cohort A, 1977–1995 (pre-immunomodulator/biological era); cohort B, 1996–2008 (immunomodulator era); and cohort C, 2008–2020 (biological era). 

### 2.2. Data Collection

The data was collected prospectively by physicians using unified case record forms. Patient data were collected from five general hospitals and gastroenterology outpatient clinics, each staffed by at least one gastroenterologist or internist with a special interest in gastroenterology in Veszprém province. The majority of patients were monitored at the Csolnoky F. Province Hospital, a referral center for IBD patients.

Furthermore, all healthcare data of Hungarian residents has been available on the online Hungarian Health Database cloud (EESZT) since 2018. The provincial IBD register data was centralized in Veszprém. Both in-hospital and outpatient records were collected and comprehensively reviewed at diagnosis and during follow-up at least yearly. Due to Hungarian health authority regulations, a follow-up visit at a specialized gastroenterology center is mandatory for all IBD patients at least every 6 months. Otherwise, the conditions of the health insurance policy change, and they forfeit their ongoing subsidized therapy.

Data were collected on patient baseline demographics, including age, gender, age of onset, date of IBD diagnosis, family history of IBD, presence of extra-intestinal manifestations (EIMs), smoking status at diagnosis, history of appendectomy before the diagnosis of IBD, use of oral contraceptives, and socioeconomic risk factors, including living area, type of employment or occupation, and education. The study questionnaire is shown in the supplementary Table S1. The disease phenotype of CD (age at onset, duration, location, and behaviors) and disease extension at diagnosis of UC were determined according to the Montreal Classification [32]. The patients in the early cohort were retrospectively reclassified after the Montreal classification became available. The disease severity of UC was assessed by the partial Mayo score (pMayo) [33].

### 2.3. Definition of Study Outcomes 

Smoking status at diagnosis was investigated by reviewing medical records at diagnosis. Active smoking was defined as having smoked at least 7 cigarettes per week for at least 6 months at diagnosis. Ex-smoking was defined as complete abstinence for at least 1 year before diagnosis [34]. Current use of oral contraceptives was defined as use within one month of the date of diagnosis; previous use was defined as use that stopped at least one month before the date of diagnosis. Appendectomy was included in the analyses only when the date of the operation showed that it was performed before a clear diagnosis of IBD. 

In this cohort, we divided patients’ education into three levels; primary school, high school or college, and university level, using the highest education level when the patient had received the diagnosis. Types of employment were categorized into physical workers, referring to jobs that mostly comprise manual labor or non-skilled workers (such as agriculture, factories, construction, cleaning, and service workers), and skilled workers, referring to jobs that may require some education/training or technical knowledge (e.g., health care practitioners, engineers, lawyers, business/financial, office workers). The living area was divided into urban areas (towns and capital) and rural areas (large and small villages) according to the geographical region in Veszprem county, using the classification from the Hungarian administrative statistics database [35].

### 2.4. Statistical Analysis 

Continuous variables were presented as medians with an interquartile range (IQR), while categorical variables were shown as numbers with percentages. The t-test or Mann–Whitney U test was used to compare continuous variables, and the χ2 test or Fisher’s exact test was used to compare categorical variables, as appropriate. A *p*-value of <0.05 was regarded as statistically significant. Statistical analyses were performed using the SPSS software version 20.0 (Chicago, IL, USA).

### 2.5. Ethical Considerations 

The study was approved by the Csolnoky F. Province Hospital Institutional Committee of Science and Research Ethics (193/2004, 0712/2009, and 2/2021). 

## 3. Results

### 3.1. Baseline Characteristics of the Study Population 

A total of 2240 patients were diagnosed with IBD (CD 38.8%, UC 61.2%), and 51.2% were men. The median age at diagnosis was 35 years (IQR, 29–49). In the total inclusion period, 459 were in cohort A (1977–1995), 984 were in cohort B (1996–2008), and 797 were in cohort C (2009–2020). Table 1 summarizes the demographic and clinical characteristics of patients with IBD in the cohorts. 

Over time, a higher proportion of patients diagnosed at ages above 40 years was observed in UC (*p* = 0.030) and CD (*p* = 0.039). The time interval from the onset of IBD symptoms to the definite diagnosis of IBD was significantly shorter in cohort C compared to cohorts A and B in both UC (*p* < 0.001) and CD (*p* = 0.002). There was no significant difference in the proportion of patients with a positive familial anamnesis of IBD across the three cohorts in both UC (*p* = 0.584) and CD (*p* = 0.246).

### 3.2. The Trends of Environmental and Socioeconomic Factors in Patients with UC 

The trends of environmental and socio-economic factors in patients with UC are shown in Table 2. At the time of diagnosis, the proportions of patients with active smoking were not significantly different (*p* = 0.916). Similarly, the proportions of ex-smokers remained unchanged (*p* = 0.913), and rates of current oral contraceptive use were stable (*p* = 0.175). However, the proportion of patients who underwent appendectomy before the diagnosis of UC significantly decreased over time: 6.3%, 5.5%, and 2.3% (*p* = 0.015). 

There were no significant changes in the socio-geographical distribution of UC patients in the living area in childhood (urban area vs. rural area, *p* = 0.309). However, a greater percentage of UC patients achieved high school or college as the highest education level (*p* < 0.001), and an increased proportion of skilled workers was observed over time (*p* = 0.027).

### 3.3. The Trends of Environmental and Socioeconomic Factors in Patients with CD

The trends of environmental and socio-economic factors in CD patients are shown in Table 3. There was a significant decrease in the proportions of CD patients with active smoking at diagnosis: 60.2%, 49.9%, and 38.5% in cohorts A, B, and C, respectively (*p* < 0.001). In addition, the rates of appendectomy prior to the diagnosis were significantly decreased: 27.3%, 14.2%, and 6.9% in cohorts A, B, and C, respectively (*p* < 0.001). In contrast, the proportion of patients with current oral contraceptive use did not show significant differences amongst the cohorts (*p* = 0.774).

Similar to the patients with UC, there were no significant differences in the living area (urban vs. rural) of CD patients (*p* = 0.636). Over time, fewer percentages of CD patients reported primary school (*p* < 0.001) but greater percentages of patients reported high school or college (*p* = 0.002) as the highest education level. However, there were no significant differences in the proportions of physical workers and skilled workers among CD patients between the cohorts (*p* = 0.454).

### 3.4. Differences in the Trend of Environmental and Socioeconomic Factors between the Incident UC and CD Patients 

**Figure 1:**
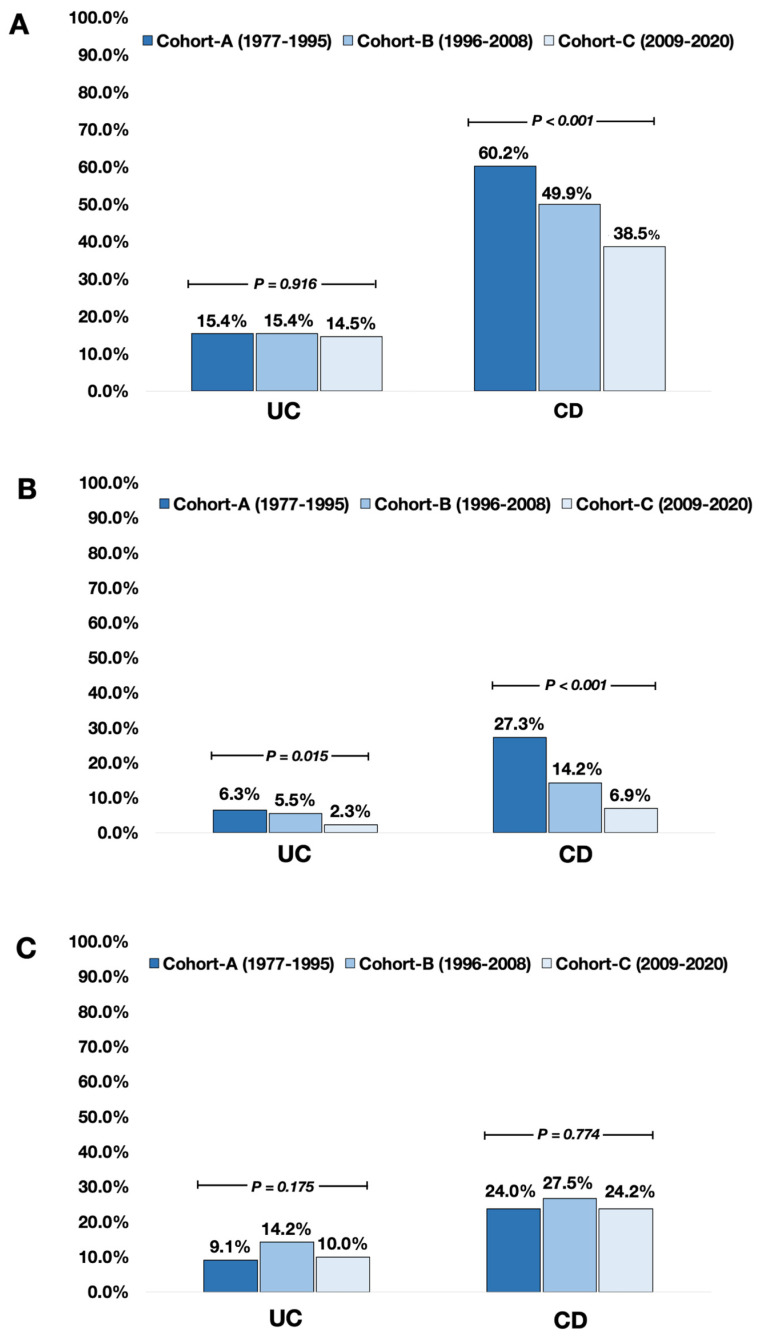
The trend of environmental and socioeconomic risk factors in the incident UC and CD patients. Note: (**A**) active smoking at diagnosis, (**B**) appendectomy before diagnosis, (**C**) oral contraceptive use at diagnosis.

**Figure 2:**
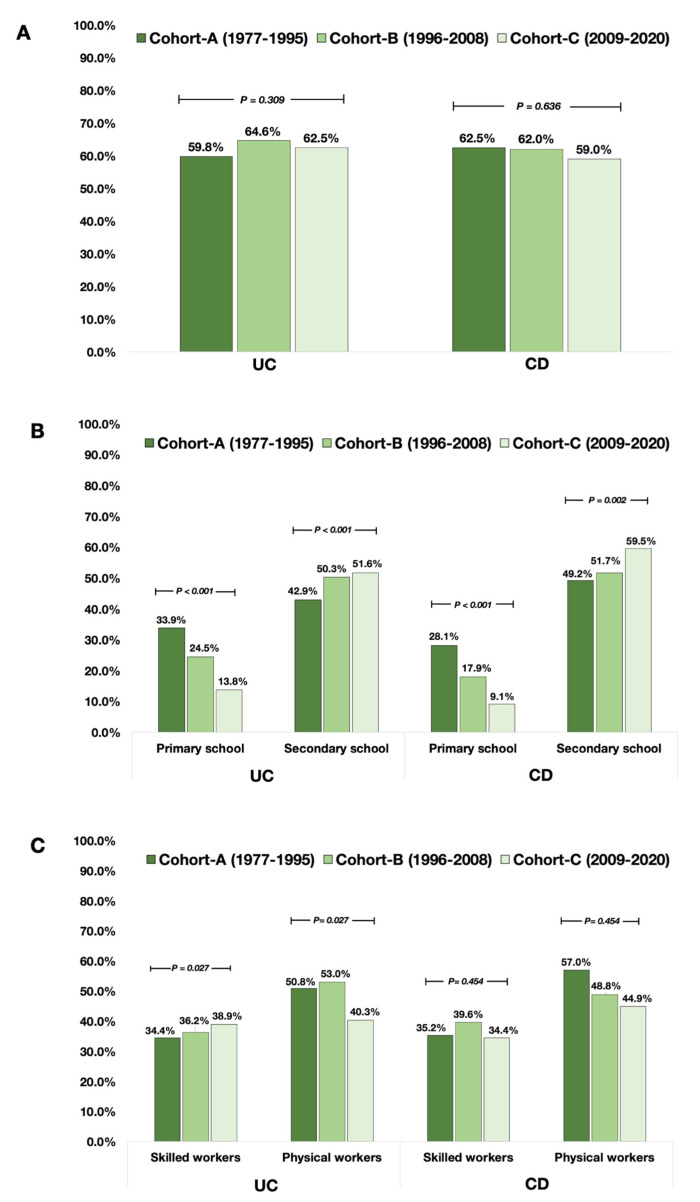
The trend of socioeconomic risk factors in the incident UC and CD patients. Note: (**A**) urban living area, (**B**) the highest education level (primary vs. secondary school or above) and (**C**) type of occupation (skilled vs. physical workers).

In the overall cohort, the proportion of patients with active smoking at diagnosis (46.7% vs. 15.1%, *p* < 0.001), the rates of appendectomy before diagnosis (13.1% vs. 4.7%, *p* < 0.001) and oral contraceptive use (25.0% vs. 11.6%, *p* < 0.001) were significantly higher in patients with CD compared to UC, respectively. However, there were no significant differences in the living area (urban area; UC 62.7% vs. CD 60.8%, *p* = 0.326) and types of employment (physical worker; UC 48.4% vs. CD 48.4%, *p* = 0.968). In addition, the proportion of patients who had high school or college degrees as their highest education level was higher in CD than in UC. (54.6% vs. 48.9% vs. *p* = 0.001). The differences in environmental and socioeconomic factors between UC and CD are detailed in Table 4 and Supplemental Table S2. The trends of key environmental risk factors in UC and CD are shown in Figure 1. The trend of socio-economic factors is shown in Figure 2.

## 4. Discussion

In this population-based inception cohort of IBD patients, we demonstrated the time-trend changes in key environmental and socioeconomic risk factors associated with developing IBD over recent decades and therapeutic eras. Despite the increased incidence of IBD in the last several decades, the rates of active smoking at diagnosis of CD patients, as one of the well-known environmental factors, have significantly decreased over time. The appendectomy rates prior to diagnosis in UC and CD declined with time. However, there were no significant differences in trends of oral contraceptive use and no major changes in trends of socio-geographic and educational factors in the IBD population. 

Smoking has been described as an environmental risk factor in IBD since the 1980s. A recent meta-analysis reported that active smoking at diagnosis was associated with an increased risk of CD (OR, 1.76; 1.40–2.22) and a decreased risk of UC (OR, 0.58; 0.45–0.75) compared with non-smokers [24]. Former smoking also increases the risk of UC (OR, 1.79; 95% CI, 1.37–2.34) [10]. A few prospective studies, however, found no association between smoking and CD in the Asian and Jewish populations [36,37]. This present population-based cohort showed that the trends of active smoking in the CD population significantly decreased over time, from 60.2% in the early cohort (1977–1995) to 38.5% in the most recent cohort (2009–2020). The proportion of ex-smokers in the CD population has significantly increased, from 6.2% to 17.4%. Of note, smoking habits in the general population were relatively stable during the study period, with a prevalence of active smokers of 33–46% among adults [38]. Thus, the rates of active smoking in CD reported in the most recent cohort (2009–2020) were close to the rates in the general population. In contrast, the prevalence of active smokers in UC was significantly lower than in CD (15.1% vs. 46.7%) or among the general population, and the rates remained stable in UC patients. 

Earlier data from our population-based cohort demonstrated that the effect of smoking on IBD was linked to gender and age at diagnosis and was most prevalent in young adults, with no significant association in either pediatric or elderly patients [34]. In addition, the effect of smoking can be influenced by genetic and ethnicity factors, and the risk of smoking in IBD was only observed in non-Jewish white individuals [24]. It is important to note that Hungary has a genetically heterogeneous population, with influences from both Asian populations (dating back to almost two centuries of Turkish occupation) and West European populations (repopulation by Slavonic and German ethnic groups after the Turkish occupation ended). As a result, findings on environmental and socio-economic factors could be relevant for a diverse population. 

We highlighted that smoking as one of the well-known risk factors in developing CD has become less important in the present study and cannot explain the changes in the incidence of CD over the last several decades, given that the rates of smoking have significantly decreased over time, despite the increased incidence and prevalence of IBD. Furthermore, the decreased smoking prevalence in UC compared to the background population was a stable phenomenon [30]. 

A previous meta-analysis found that appendectomy was linked with an increased risk of CD (relative risk (RR), 1.61; 1.28–2.02) at 1–4 years following an appendectomy [12]. However, after 5 years, the risk had returned to baseline. Similarly, a large Swedish–Danish cohort also showed that the risk was highest in the first 6 months (standard incidence ratio, SIR; 8.7) and then diminished rapidly until it reached background levels at 5–10 years [39]. Thus, the risk may result from unnecessary appendectomies performed on patients with incipient CD who present with symptoms similar to appendicitis [12]. In the present cohort, a significant decrease in rates of appendectomy prior to diagnosis of CD, from 27.3% in the early cohort to 6.9% in the most recent cohort, indicates that an appendectomy was unlikely to be associated with the increase in the incidence of CD [39,40]; the trends in appendectomy rates significantly decreased over time, particularly in the most recent cohort (2009–2020), while CD has become more common. 

On the other hand, more accurate diagnostic tools could have avoided unnecessary appendectomies in newly diagnosed CD patients. In contrast to CD, appendectomy has been shown to be a strong protective factor in UC (OR, 0.39; 0.29–0.52) [24,41,42]. Despite this, overall rates of appendectomy were low; only 4.7% of UC patients underwent appendectomy prior to diagnosis, with a declining trend over time. Thus, our findings suggest that appendectomy is not associated with the development of IBD.

The association between oral contraceptive use and increased risk of IBD has been reported in CD (OR, 1.25; 1.05–1.48) and UC (OR, 1.28; 1.08–1.52) [24]. However, a large prospective cohort found an increased risk solely in CD and a non-significant risk in UC [11]. The present cohort demonstrated a significantly higher proportion of patients with oral contraceptive use in CD compared to UC (25.0% vs. 11.6%), suggesting a possible association between oral contraceptive use and an increased risk of CD in female patients. Additionally, the trends of oral contraceptive use have remained unchanged over time in both CD and UC.

The living environment in the childhood period has been reported to be associated with developing IBD, as highlighted by the hygiene hypothesis. Urban living was associated with both the risk of CD (incidence rate ratio (IRR), 1.42; 1.26–1.60); and UC (IRR, 1.17; 1.03–1.32) [16,18]. A large meta-analysis showed that living near farm animals had a protective role in CD (OR, 0.46; 0.20–0.72) and UC (OR, 0.44; 0.14–0.74) [19]. In the present study, the distribution of urban living in the incident IBD population was constant over time in both UC and CD. 

The occupational risk factors were assessed in the present cohort as they linked to individual daily physical activity and the development of IBD [23,43]. In a German cohort from 1982–88, occupations involving physically demanding, open-air, and unskilled work decreased the risk of developing IBD, while being exposed to air-conditioned artificial working conditions, or extended/irregular shifts increased the risk of IBD [23]. In CD, professional workers (OR 4.2) and administrative staff (OR 5.5) were at increased risk. In contrast, working in agriculture, forestry, animal husbandry, fisheries and water conservancy decreased the risk (OR 0.09) [43]. Moreover, several studies showed that increased physical activity decreases the risk of CD (RR, 0.63; 0.50–0.79) but not UC (RR, 0.82; 0.68–1.00) [21,22,36]. In the present cohort, the trends of occupational factors were not significantly changed in the CD patients. Still, numerically fewer physical workers were observed in UC (50.8% in the early cohort and 40.3% in the most recent cohort).

Our study also evaluated the education factors, which might be linked to individual self-care and personal hygiene. The association between education and IBD could also be interpreted as a chronic illness caused by IBD that might reduce the patient’s ability to complete their education during their childhood [44]. In the present cohort, we failed to show a significant undereducation rate. In fact, a greater percentage of IBD patients had completed at least secondary school, the highest education level at diagnosis in both UC and CD. The percentage of patients who completed at least secondary school was comparable to the general population aged 25–64 years in Hungary [45]. Of course, college or university education level could not be evaluated at diagnosis in adolescent patients in our cohort. 

The proportions of patients with UC and CD with first-degree relatives who had IBD were relatively stable in this present cohort. Despite the fact that the prevalence of multiple key environmental risk factors for IBD has changed over time, their trends are sometimes contrary to what would be expected for IBD, and they cannot clearly explain the rapidly increasing incidence of IBD. Thus, we hypothesize that other possible factors which were not captured can influence the pathogenesis of IBD, particularly the gut microbiome and dietary intake [46]. Of note, fast-food chains were introduced in Hungary in the mid-1980s. Moreover, the traditional methods of food preservation were replaced by refrigeration in the early 1970s. Hence, the effect of dietary factors may be much more pronounced than previously recognized. Similarly, air pollution or chemical mimicry between plastic and macro-molecules could play a role in the acceleration of IBD incidence. Furthermore, a recent large population-based study of IBD patients revealed that increased prevalence of IBD may be linked to factors not traditionally associated with IBD, such as loss of diversity and geosocial features [47]. Other factors, such as prenatal smoking exposure, bronchial hyperreactivity, and stressful life events, may also be linked to an increased risk of IBD [15]. 

The main strengths of the present study are that we were able to include a large, long-term prospective population-based inception cohort of incident IBD patients in well-defined geographical areas representing multiple therapeutic eras. Thus, we were able to assess the long-term trends of the environmental and socioeconomic factors according to the different decades of IBD diagnosis. Our study used stringent diagnostic criteria to identify accurate IBD cases and captured extensive data using harmonized case record forms that were regularly collected by medical professionals.

There are some limitations to our study. First, although we did not compare the trends of risk factors with a control population, our study provides the first data on trends and the magnitude of environmental risk factors associated with IBD in a large, well-established population-based cohort. Second, we did not collect some details regarding smoking habits and oral contraceptive use, such as quantity or duration of exposure. Similarly, the time interval to IBD diagnosis after an appendectomy was not collected. Third, we did not capture other potential factors that can influence the development of IBD, such as breastfeeding, tonsillectomy, antibiotics, childhood infections or vaccination, and dietary factors [48]. Lastly, we divided the patients into three cohorts based on the different therapeutic eras of their IBD diagnosis. However, it was not certain that all patients were treated with the maximum therapeutic step as defined in the given period.

## 5. Conclusions

In conclusion, this is one of the first long-term, population-based inception cohorts that investigated the association between time trends of known environmental and socioeconomic factors and IBD incidence over a 40-year period. The interaction between environmental factors and the development of IBD is complex. Smoking, oral contraceptive use (in females), appendectomy, and socioeconomic/hygiene factors may interact differently with host genetic factors in each patient. The present study has not identified major changes in environmental factors that would explain the increase in the incidence of IBD over the four decades of the different therapeutic eras of IBD. In contrast, smoking at the time of diagnosis became less prevalent in CD patients. Indirectly, our findings support the idea that dietary factors and microelements may play a larger role in the pathogenesis of IBD than previously thought.

## Figures and Tables

**Table 1:** Demographic and clinical characteristics of 2240 incident IBD patients stratified by era of diagnosis (cohort A vs. cohort B vs. cohort C) and by type of disease (UC vs. CD).

Characteristics	Cohort-A, 1977–1995 (n = 459)		Cohort-B, 1996–2008 (n = 984)		Cohort-C, 2009–2020 (n = 797)		*p*-Value *
	UC (n = 331)	CD (n = 128)	UC (n = 605)	CD (n = 379)	UC (n = 434)	CD (n = 363)	
Male gender	163 (49.2%)	53 (41.1%)	324 (53.6%)	186 (49.1%)	222 (51.2%)	198 (54.5%)	0.431 (UC) 0.032 (CD)
Median age at diagnosis, years	37.0	31.0	38.0	29.0	38.0	34.0	0.515 (UC)
IQR, years	(29.0–49.0)	(25.0–39.0)	(26.0–51.0)	(27.0–52.0)	(28.0–55.0)	(25.0–47.0)	<0.001 (CD)
Mean interval from onset of symptoms to IBD diagnosis, years (SD)	1.82 (4.35)	1.95 (3.63)	0.81 (2.57)	1.15 (2.03)	0.42 (1.11)	1.05 (2.45)	<0.001 (UC) 0.002 (CD)
Age group at diagnosis							
17–40 years	206 (62.2%)	103 (80.5%)	324 (53.6%)	293 (77.0%)	237 (54.6%)	254 (69.7%)	0.030 (UC)
>40 years	125 (37.8%)	25 (19.5%)	281 (46.4%)	86 (22.7%)	197 (45.4%)	106 (29.2%)	0.039 (CD)
First-degree relatives with IBD	32 (9.7%)	24 (18.8%)	69 (11.4%)	60 (15.8%)	52 (12.0%)	47 (12.9%)	0.584 (UC) 0.246 (CD)
Disease location (UC/CD) ^†^							
- E1/L1	69 (20.8%)	39 (30.5%)	168 (27.8%)	101 (26.7%)	94 (21.7%)	127 (35.0%)	0.002 (UC)
- E2/L2	183 (55.3%)	44 (34.4%)	296 (48.9%)	143 (37.7%)	202 (46.5%)	93 (25.6%)	0.008 (CD)
- E3/L3	79 (23.9%)	45 (35.1%)	141 (23.3%)	135 (35.6%)	138 (31.8%)	143 (39.4%)	
Disease severity at diagnosis UC							
- Mild-Moderate	206 (62.2%)		324 (53.6%)		237 (54.6%)		0.004
- Severe ^‡^	125 (37.8%)		281 (46.4%)		197 (45.4%)		
Behaviour of CD							
- Non-stricture		60 (46.9%)		236 (62.2%)		212 (58.4%)	
- Stricture		26 (20.3%)		73 (19.3%)		70 (19.3%)	0.006
- Penetrating		26 (20.3%)		53 (14.0%)		66 (18.2%)	
- Stricture with penetrating		16 (12.5%)		17 (4.5%)		15 (4.1%)	
Upper GI involvement in CD		1 (0.8%)		8 (2.1%)		18 (5.0%)	0.021
Perianal disease in CD		32 (25.0%)		65 (17.2%)		39 (10.7%)	0.007

Abbreviations: CD, Crohn’s disease; CI, Confidence interval; IBD, Inflammatory bowel disease; IQR, Interquartile range; SD, standard deviation; UC, Ulcerative colitis. Note: ^†^ Disease location was classified using Montreal classification: E1, proctitis; E2, left-sided colitis; E3, extensive colitis; L1, terminal ileal; L2, colonic; L3 ileo-colonic. ^‡^ Severe disease activity was defined as a partial Mayo score of 7–9. * The *p*-value indicates whether there is a statistically significant difference among the three cohorts: A, B and C.

**Table 2:** Trend of environmental factors of 1370 incident UC patients stratified by the eras of UC diagnosis.

Factors	Total (n = 1370)	Cohort-A, 1977–1995 (n = 331)	Cohort-B, 1996–2008 (n = 605)	Cohort-C, 2009–2020 (n = 434)	*p*-Value *
**Environmental factors**					
Smoking status at diagnosis					
Non-smoking	905 (66.1%)	219 (66.2%)	395 (65.3%)	291 (67.1%)	0.839
Active smoking	207 (15.1%)	51 (15.4%)	93 (15.4%)	63 (14.5%)	0.916
Ex-smoking	258 (18.8%)	61 (18.4%)	117 (19.3%)	80 (18.4%)	0.913
Appendectomy before diagnosis UC	64 (4.7%)	21 (6.3%)	33 (5.5%)	10 (2.3%)	0.015
Oral contraceptive use					
Current use	76/657 (11.6%)	15/165 (9.1%)	40/281 (14.2%)	21/211 (10.0%)	0.175
Previous use	190/657 (28.9%)	42/165 (25.5%)	86/281 (30.6%)	62/211 (29.4%)	0.503
% only in female					
**Socioeconomic factors**					
Area of living					
Urban area	859 (62.9%)	198 (59.8%)	391(64.8%)	270 (62.5%)	0.309
Rural area	507 (37.1%)	133 (40.2%)	212 (35.2%)	162 (37.5%)	0.309
Highest education level					
Primary school	320 (23.4%)	112 (33.9%)	148 (24.5%)	60 (13.8%)	<0.001
High school/college	670 (48.9%)	142 (42.9%)	304 (50.3%)	224 (51.6%)	<0.001
University	230 (16.8%)	59 (17.8%)	109 (18.1%)	62 (14.3%)	0.852
No data	149 (10.9%)	18 (5.4%)	43 (7.1%)	88 (20.3%)	NA
Type of employment					
Skilled worker	502 (36.7%)	114 (34.4%)	219 (36.2%)	169 (38.9%)	0.027
Physical worker	663 (48.4%)	168 (50.8%)	320 (53.0%)	175 (40.3%)	0.027
Unemployed/no data	204 (14.9%)	49 (14.8%)	65 (10.8%)	90 (20.8%)	NA

Abbreviations: UC, Ulcerative colitis; NA, not applicable. Note: * The *p*-value indicates whether there is a statistically significant difference among the three cohorts: A, B and C.

**Table 3:** Trend of environmental factors of 870 incident CD patients stratified by the eras of CD diagnosis.

Factors	Total (n = 870)	Cohort-A, 1977–1995 (n = 128)	Cohort-B, 1996–2008 (n = 379)	Cohort-C, 2009–2020 (n = 363)	*p*-Value *
**Environmental factors**					
Smoking at diagnosis					
Non-smoking	357 (41.0%)	43 (33.6%)	154 (40.6%)	160 (44.1%)	0.114
Active smoking	406 (46.7%)	77 (60.2%)	189 (49.9%)	140 (38.5%)	<0.001
Ex-smoking	107 (12.3%)	8 (6.2%)	36 (9.5%)	63 (17.4%)	<0.001
Appendectomy before diagnosis CD	114 (13.1%)	35 (27.3%)	54 (14.2%)	25 (6.9%)	<0.001
Contraceptive use					
Current use	111/433 (25.6%)	18/75 (24.0%)	53/193 (27.5%)	40/165 (24.2%)	0.774
Previous use	132/433 (30.4%)	24/75 (32.0%)	56/193 (29.0%)	52/165 (31.5%)	0.798
% only in female					
**Socioeconomic factors**					
Area of living					
Urban area	529 (60.8%)	80 (62.5%)	235 (62.0%)	214 (59.0%)	0.636
Rural area	341 (39.2%)	48 (37.5%)	144 (38.0%)	149 (41.0%)	0.636
Highest education level					
Primary school	137 (15.7%)	36 (28.1%)	68 (17.9%)	33 (9.1%)	<0.001
High school/college	475 (54.6%)	63 (49.2%)	196 (51.7%)	216 (59.5%)	0.002
University	151 (17.4%)	26 (20.3%)	67 (17.7%)	58 (16.0%)	0.887
No data	107 (12.3%)	3 (2.4%)	48 (12.7%)	56 (15.4%)	NA
Type of employment					
Skilled workers	320 (36.9%)	45 (35.2%)	150 (39.6%)	125 (34.4%)	0.454
Physical workers	421 (48.5%)	73 (57.0%)	185 (48.8%)	163 (44.9%)	0.454
Unemployed/no data	129 (14.6%)	10 (7.8%)	44 (11.6%)	75 (20.7%)	NA

Abbreviations: CD, Crohn’s disease; NA, not applicable. Note: * The *p*-value indicates whether there is a statistically significant difference among the three cohorts: A, B and C.

**Table 4:** Differences in environmental and socio-economic factors between the incident UC and CD patients stratified by the eras of diagnosis.

Factors	UC (n = 1370)	CD (n = 870)	*p*-Value
Acute smoking at diagnosis	207 (15.1%)	406 (46.7%)	<0.001
Appendectomy before diagnosis	64 (4.7%)	114 (13.1%)	<0.001
Contraceptive use at diagnosis % only in female	76/657 (11.6%)	111/444 (25.0%)	<0.001
Physical workers	663 (48.4%)	421 (48.4%)	0.968
Urban living area	859 (62.7%)	529 (60.8%)	0.326
Primary school	320 (23.4%)	137 (15.7%)	<0.001
High school/college	670 (48.9%)	475 (54.6%)	0.001
University	230 (16.8%)	151 (17.4%)	0.639

## Data Availability

The main data are given in this article. The data are available from the corresponding author upon request.

## Supplementary Materials

The following supporting information can be downloaded at: https://www.mdpi.com/article/10.3390/jcm12083026/s1. Table S1: The study questionnaire; Table S2: The differences in environmental and socioeconomic factors between UC and CD.
