# Supplementary material for: Time Trends of Environmental and Socioeconomic Risk Factors in Patients with Inflammatory Bowel Disease over 40 Years: A Population-Based Inception Cohort 1977–2020
Source: J Clin Med. 2023 Apr 21;12(8):3026. doi: 10.3390/jcm12083026 (PMC10147007; doi:10.3390/jcm12083026)
Supplement: Supplementary file 1 [file jcm-12-03026-s001.zip › Table S1.pdf]

Supplementary Table S1

Epidemiologiai vizsgálat. Kérdőív. Crohn-betegség

„A gyulladásos bélbetegségek epidemiológiájának vizsgálata gyulladásos bélbetegekben Veszprém megyében."

Betegazonosító adatok és klinikai jellemzők
Betegazonosító:________________________

**Életkor a betegség kezdetekor**	év			
**Betegség fennállásának ideje**	év (diagnóziskor 0 év)			
**Beteg életkora most**	év			
**Nem**	F	N		
**Lakohely**	varos	falu		
**Iskolazottsag**	alapfokú	középfokú	egyetem/főiskola	
**Munkakör**	fizikai	szellemi	tanuló	
**Antikoncipiens (ha nő)**	igen	nem		
**Appendectomia a diagnozis előtt**	igen	nem		
**Familiaris anamnezis**	igen	Nem		
	UC		CD	
	Elsőfokú	Másodfokú	Elsőfokú	Másodfokú
**Dohányzás a diagnoziskor**	<15	≥15	régen	nem
**Dohányzás változás**	igen	nem	mikor?	most mennyi?
**Lokalizáció**	L1	L2	L3	L4
**Viselkedése, típusa**	B1	B2	B3	B3-4
**Viselkedés változása**	igen	nem	mikor?	Új behavior:
**Aktivitás (CDAI, HBI)**		Ekkor CRP-thrombocyta szám?		
**Perianalis szövődmény**	igen	Nem		
**Gyakori relapsus**	igen	Nem	évente hány?	
**Extraintestinalis: arthritis**	igen	Nem		
**Peripheriás**	igen	Nem		
**Axiális (sacroileitis, SNSA)**	igen	Nem		
**Extraintestinalis: máj**	igen	Nem		
**PSC, Pericholangitis**	igen	Nem		
**AIH**	igen	Nem		
**NAFLD**	igen	Nem		
**Extraintestinalis: szem**	igen	Nem	Melyik?	
**Extraintestinalis: bőr**	igen	Nem	Melyik?	
**Terápia**				
**ASA**	salazopyrin	mesalasin	Egyéb:	
**Mennyi ideig?**	Év			
**Milyen dózisban**	<2 g		≥2 g	
**Steroid**	igen	Nem	**Ha igen:** refracter	**Ha igen:** dependens
**Mennyi ideig?**	Év			
**Azathioprin**	igen	Nem	**Ha igen:** refracter	**Ha igen:** dependens
**Mennyi ideig?**	Év			
**Dózis?**	<50 mg	50-75 mg	≥100 mg	
**Egyéb immunszupresszív**	nem	**Igen:** cyclosporin	**Igen:** methothrexat	
**Biologiai terápia? mikor?**	Igen	Nem	**Ha igen:** infliximab	Egyéb:
**kezelés hatékonysága**	remisszió	response	nem reagált	szövődmény

**Resectios műtétek száma**	0	1	2-3	>3
**Műtétig eltelt idő (hónap a diagnozistól)**	első	második		

Értelmezés: Betegazonosító jel: centrum betűjele / sorszám az adott centrumban / név kezdőbetűk, Pl: **V/1/NI** (Veszprém/1. beteg/Nagy István)
Lokalizáció: L1: ileum L2: colon L3: ileocolicus L4: felső GI tractus
Behavior: B1: gyulladásos, B2: stenotisáló, B3: penetráló B3-4: stenotisáló és penetráló
Gyakori relapsus: 2 éven belül 2x (CDAI >150),
Extraintest. arthritis: axialis (sacroileitis, SNSA) és peripheriás („colitises”) arthritisek (P1= nagy izület (térd, váll), P2 = kis izület (kéztő, stb.), **Extraintest. szem:** uveitis, iritis, conjunctivitis, **Extraintest. bőr:** erythema nodosum, pyoderma gangraenosum, **Extraintestinális máj**: PSC, pericholangitis, AIH, NAFLD

Epidemiologiai vizsgálat. Kérdőív. Colitis ulcerosa

„A gyulladásos bélbetegségek epidemiológiájának vizsgálata gyulladásos bélbetegekben Veszprém megyében.”

Betegazonosító adatok és klinikai jellemzők

Betegazonosító:________________________

**Életkor a betegség kezdetekor**		év			
**Betegség fennállásának ideje**		év (diagnóziskor 0 év)			
**Beteg életkora most**		év			
**Nem**		F	N		
**Lakohely**		varos	falu		
**Iskolazottsag**		alapfokú	középfokú	egyetem/főiskola	
**Munkakör**		fizikai	szellemi	tanuló	
**Antikoncipiens (ha nő)**		igen	nem		
**Appendectomia a diagnozis előtt**		igen	nem		
**Familiaris anamnezis**		igen	Nem		
		UC		CD	
		Elsőfokú	Másodfokú	Elsőfokú	Másodfokú
**Dohányzás a diagnoziskor**		<15	≥15	régen	nem
**Dohányzás változás**		igen	nem	mikor?	most mennyi?
**Lokalizáció**	Proctitis	Procto-sigmoiditis	Bal oldali colitis	Subtotalis colitis	Pancolitis
**Lefolyás**		Remittáló		chr. continuus	
**Aktivitás (Mayo-TW index)**				Ekkor CRP-thrombocyta szám?	
		Gyakori relapsus	Ritka relapsus	évente hány?	
**Extraintestinalis: arthritis**		igen	Nem	Egyéb:	
**Peripheriás**		igen	Nem		
**Axiális (sacroileitis, SNSA)**		igen	Nem		
**Extraintestinalis: máj**		igen	Nem		
**PSC, Pericholangitis**		igen	Nem		
**AIH**		igen	Nem		
**NAFLD**		igen	Nem		
**Extraintestinalis: szem**		igen	Nem	Melyik?	
**Extraintestinalis: bőr**		igen	Nem	Melyik?	
**Terápia**					
**ASA**		salazopyrin	mesalasin	Egyéb:	
**Mennyi ideig?**		Év			
**Milyen dózisban**		<2 g		≥2 g	
**Steroid**		igen	Nem	**Ha igen:** refracter	**Ha igen:** dependens
**Mennyi ideig?**		Év			
**Azathioprin**		igen	Nem	**Ha igen:** refracter	**Ha igen:** dependens
**Mennyi ideig?**		Év			
**Dózis?**		<50 mg	50-75 mg	≥100 mg	
**Biologiai terápia? mikor?**		Igen	Nem	**Ha igen:** infliximab	Egyéb:
**kezelés hatékonysága**		remisszió	response	nem reagált	szövődmény
**Egyéb immunszupresszív**		nem	**Igen:** cyclosporin	**Igen:** methothrexat	**Igen:** egyéb
**Colectomia**		igen	Nem		
**Mikor (hónap a diagnozistól)**					

Értelmezés: Betegazonosító jel: centrum betűjele / sorszám az adott centrumban / név kezdőbetűk, Pl: **V/1/NI** (Veszprém/1. beteg/Nagy István)
Gyakori relapsus: 2 éven belül 2x (CDAI >150),
Extraintest. Arthritis: axialis (sacroileitis, SNSA) és peripheriás („colitises”) arthritisek (P1= nagy izület (térd, váll), P2 = kis izület (kéztő, stb.), **Extraintest. Szem:** uveitis, iritis, conjunctivitis, **Extraintest. Bőr:** erythema nodosum, pyoderma gangraenosum, **Extraintestinális máj**: PSC, pericholangitis, AIH, NAFLD
