# Supplementary material for: Time Trends of Environmental and Socioeconomic Risk Factors in Patients with Inflammatory Bowel Disease over 40 Years: A Population-Based Inception Cohort 1977–2020
Source: J Clin Med. 2023 Apr 21;12(8):3026. doi: 10.3390/jcm12083026 (PMC10147007; doi:10.3390/jcm12083026)
Supplement: Supplementary file 1 [file jcm-12-03026-s001.zip › Table S2.pdf]

Supplemental Table S2 Differences in environmental and socio-economic factors between the incident UC and CD patients stratified by the eras of diagnosis

Factors	Cohort A			Cohort B			Cohort C		
	**UC (n=331)**	**CD (n=128)**	***p-value***	**UC (n=605)**	**CD (n=379)**	***p-value***	**UC (n=434)**	**CD (n=363)**	***p-value***
**Current Smoking at diagnosis**	51 (15.4%)	77 (60.2%)	*<0.001*	93(15.4%)	189 (49.9%)	*<0.001*	63(14.5%)	140(38.6%)	*<0.001*
**Appendectomy before diagnosis**	21 (6.3%)	35 (27.3%)	*<0.001*	33 (5.5%)	54 (14.2%)	*<0.001*	10(2.3%)	25(6.9%)	*0.003*
**Current Contraceptive use at diagnosis** **% counted only in female*	15/165(9.1%)	18/76(23.7%)	*0.004*	40/281(14.2%)	53/199(26.6%)	*0.001*	21/211(10.0%)	40/169(23.7%)	*<0.001*
**Physical workers**	168 (50.8%)	73 (57.0%)	*0.737*	320 (52.9%)	185(48.8%)	*0.232*	175(40.3%)	163 (44.9%)	*0.173*
**Urban living area**	198 (59.8%)	80 (62.5%)	*0.670*	391 (64.6%)	235 (62.0%)	*0.376*	270(62.2%)	214 (59.0%)	*0.308*
**Primary school**	112 (33.8%)	36 (28.1%)	*0.181*	148 (24.5%)	68 (17.9%)	*0.016*	60(13.8%)	33 (9.1%)	*0.043*
**High school/college**	142 (42.9%)	63 (49.2%)	*0.342*	304 (50.2%)	196 (51.7%)	*0.354*	216 (51.6%)	196 (59.5%)	*0.034*
**University**	59 (17.8%)	26 (20.3%)	*0.688*	109 (18.0%)	67(17.6%)	*0.931*	62(14.3%)	58 (16.0%)	*0.477*
